# Stereotactic Body Radiotherapy for Metastatic and Recurrent Ewing Sarcoma and Osteosarcoma

**DOI:** 10.1155/2014/418270

**Published:** 2014-12-09

**Authors:** Lindsay C. Brown, Rachael A. Lester, Michael P. Grams, Michael G. Haddock, Kenneth R. Olivier, Carola A. S. Arndt, Peter S. Rose, Nadia N. Laack

**Affiliations:** ^1^Department of Radiation Oncology, Mayo Clinic, 200 First Street SW, Rochester, MN 55905, USA; ^2^Mayo Medical School, College of Medicine, Mayo Clinic, 200 First Street SW, Rochester, MN 55905, USA; ^3^Division of Pediatric Hematology/Oncology, Mayo Clinic, 200 First Street SW, Rochester, MN 55905, USA; ^4^Division of Orthopedic Oncology, Mayo Clinic, 200 First Street SW, Rochester, MN 55905, USA; ^5^Department of Orthopedic Surgery, Mayo Clinic, 200 First Street SW, Rochester, MN 55905, USA

## Abstract

*Background*. Radiotherapy has been utilized for metastatic and recurrent osteosarcoma and Ewing sarcoma (ES), in order to provide palliation and possibly prolong overall or progression-free survival. Stereotactic body radiotherapy (SBRT) is convenient for patients and offers the possibility of increased efficacy. We report our early institutional experience using SBRT for recurrent and metastatic osteosarcoma and Ewing sarcoma. *Methods*. We reviewed all cases of osteosarcoma or ES treated with SBRT between 2008 and 2012. *Results*. We identified 14 patients with a total of 27 lesions from osteosarcoma (*n* = 19) or ES (*n* = 8). The median total curative/definitive SBRT dose delivered was 40 Gy in 5 fractions (range, 30–60 Gy in 3–10 fractions). The median total palliative SBRT dose delivered was 40 Gy in 5 fractions (range, 16–50 Gy in 1–10 fractions). Two grade 2 and 1 grade 3 late toxicities occurred, consisting of myonecrosis, avascular necrosis with pathologic fracture, and sacral plexopathy. Toxicity was seen in the settings of concurrent chemotherapy and reirradiation. *Conclusions*. This descriptive report suggests that SBRT may be a feasible local treatment option for patients with osteosarcoma and ES. However, significant toxicity can result, and thus systematic study is warranted to clarify efficacy and characterize long-term toxicity.

## 1. Introduction

Osteosarcoma and Ewing sarcoma (ES) are the most common pediatric primary bone malignancies, although combined they account for fewer than 10% of pediatric malignancies [1]. While typically classified as pediatric sarcomas, osteosarcoma and ES can arise in both children and adults.

Treatment strategies are well defined for localized ES and osteosarcoma and significant improvement in outcomes has occurred over the past several decades [2]. However, the treatment of recurrent and metastatic disease remains a challenge. Distant metastases are common in patients with ES, with approximately 25% of patients presenting with distant disease [2] and an additional 20% of patients with initially localized disease developing distant metastases in the modern era [3]. Long-term event-free survival (EFS) on the order of 20–30% has been reported in metastatic ES, and aggressive local treatment of all sites of disease has been associated with significant improvement in outcomes [4].

Distant metastases are similarly common in osteosarcoma. Distant disease is present at diagnosis in approximately 10–20% of patients [6] and develops in 30–50% of patients who initially present with localized disease [5]. Ten-year survival of 24% for patients with metastatic disease at presentation has been reported [6]. Localized osteosarcoma is typically treated with chemotherapy and surgical resection; radiotherapy is reserved for rare unresectable cases. However, chemotherapy and surgery often cannot adequately address metastatic disease, and it has been demonstrated that incomplete surgical resection of metastatic disease is associated with poorer outcomes [6]. For patients with unresectable disease or in those for whom surgery will entail significant morbidity, radiotherapy may be an effective alternative strategy.

Stereotactic body radiotherapy (SBRT) is one potential treatment option for patients with metastatic or recurrent ES and osteosarcoma. SBRT is noninvasive and has a convenient fractionation schedule, minimizing chemotherapy delays. Moreover, SBRT uses strict immobilization, advanced image guidance, and sophisticated treatment planning and delivery systems, resulting in highly conformal dose distributions that allow decreases in the size of treatment volumes relative to conventional radiotherapy. This, in turn, allows for delivery of large doses of radiation per fraction and increased biologically effective doses (BED) beyond those possible with conventional treatments. In addition to theoretical use in the curative/definitive treatment of metastatic or recurrent ES and osteosarcoma, SBRT may be of value in palliating symptoms associated with disease progression, for the reasons described above.

Thus, SBRT is, theoretically, an attractive alternative to surgical resection, conventional radiation, or other palliative measures for certain patients with metastatic or recurrent osteosarcoma or ES. In the current study, we report our early institutional experience with SBRT in patients with osteosarcoma and ES.

## 2. Materials and Methods

### 2.1. Patients

With the approval of our institutional review board, we retrospectively searched our patient database for the records of patients with recurrence of or metastases from osteosarcoma or ES who were treated with SBRT at our institution from 2008 through 2012. Both adults and children with ES and osteosarcoma were included in the analysis.

The American Society for Therapeutic Radiology and Oncology and American College of Radiology have published guidelines for the performance of SBRT, in which SBRT is defined as “an external beam radiation therapy method used to very precisely deliver a high dose of radiation to an extracranial target within the body, using either a single dose or a small number of fractions [7].” In the present study, we included patients treated with 10 or fewer fractions, high dose per fraction (≥5 Gy), strict immobilization, and daily stereotactic image guidance, generally with minimal planning target volume (PTV) margin, as described below. Toxicity was graded according to the Common Terminology Criteria for Adverse Events, Version 4.0 [8]. We included patients treated with curative intent, definitive intent (i.e., with the intention of delivering an ablative dose of radiation such that subsequent treatment would not be required for the particular lesion being treated, while recognizing that progression will likely occur elsewhere), and palliative intent (i.e., with the intention of palliating pain or other symptomatologies or in order to delay symptom development). Intent of treatment was determined by the treating physician. For the purposes of description of technique, patients are considered based on intent of treatment, with patients treated with definitive and curative intent grouped together, as fractionation schemes were similar for the two groups.

Follow-up imaging was dictated by the intent of treatment. In patients undergoing curative/definitive treatment, follow-up imaging was typically performed 3 months after treatment and every 3 to 6 months thereafter. Imaging modality varied with tumor location but always included cross-sectional imaging in the form of computed tomography (CT), positron-emission tomography/CT, or magnetic resonance imaging (MRI). In patients treated with palliative intent, imaging was performed based on the presence of symptoms. For patients treated palliatively, only known local failures are presented, with no information provided regarding local control, as patients did not have routine imaging.

### 2.2. SBRT Technique

CT-based simulation was performed for all patients. Patients were immobilized for simulation and treatment in a BodyFIX immobilization device (Elekta) or thermoplastic mask. Patients expected to have significant tumor motion with respiration underwent 4-dimensional CT-based planning. Use of contrast at the time of simulation was left to the discretion of the treating radiation oncologist.

Delineation of tumor volumes and organs at risk was performed on either GE Advantage (GE Healthcare) or Eclipse (Varian Medical Systems, Inc.) workstations. Fusion with MRI and other diagnostic studies was performed at the discretion of the treating physician, when believed to aid in the definition of tumor and normal tissues. Expansions for clinical target volumes (CTV) and PTV were individualized on the basis of the clinical scenario, including tumor location and size, proximity of organs at risk, prior history of radiation, and intended dose and fractionation. For osseous lesions, CTV was typically determined by expanding the gross tumor volume by 1 cm within bone. If tumor extended into soft tissue, expansion into surrounding soft tissue by 5 mm was performed to account for possible microscopic disease. In the absence of soft tissue extension, no empiric extraosseous expansion was performed. Median expansion of CTV to PTV was 2 mm (range, 0–8 mm). For pulmonary tumors, patients underwent 4-dimensional CT simulation, allowing delineation of an internal tumor volume that accounts for tumor motion with respiration. PTV was an expansion from internal tumor volume, typically by 5 mm, with no defined CTV. TG-101 [12] dose constraints were generally used, with 10% reductions for pediatric patients (<18 years) or for patients receiving concurrent chemotherapy. PTV constraints required that 90% of the volume receive prescription dose, if critical normal tissue constraints could be met. If the normal tissue constraints could not be met, PTV coverage was sacrificed (with exceptions, as detailed in the Results section). Treatment planning was performed using Eclipse software.

All treatments were image-guided. For pulmonary lesions, daily cone-beam CT matching was performed. For osseous lesions, orthogonal kV imaging and ExacTrac (Brainlab) robotic table positioning were employed. A radiation oncologist was present at the treatment console and performed matching before treatment each day. Dose and fractionation pattern were determined by the treating physician.

### 2.3. Statistical Analysis

Statistics were performed using JMP software (SAS Institute, Inc.). Statistics were calculated from the first day of SBRT. Local failure was defined as tumor progression within the previously targeted volume.

## 3. Results

### 3.1. Treatment, Follow-Up, and Local Failure

Our search identified 14 patients with 27 osseous (*n* = 21) or pulmonary (*n* = 6) metastases from osteosarcoma (*n* = 19) or ES (*n* = 8) who received SBRT during the study period. Fourteen lesions were treated with definitive or curative intent; 13 were treated in order to palliate or delay development of symptoms. Patient and tumor characteristics for the group are shown in Table 1. Median patient age was 24 years; 6 patients were less than 18 years of age at the time of treatment. Patients were treated on consecutive weekdays. Dose selection was influenced by intention of treatment, patient, tumor, and treatment characteristics, and provider preference.

Tables 2 and 3 detail tumor and treatment characteristics for lesions treated with curative/definitive and palliative intent, respectively. Two patients were initially treated with curative intent and later with palliative intent and thus appear in both tables. Footnotes denote patient and tumor characteristics that contributed to dose selection. As noted, four thoracic spine lesions in 2 patients were treated with additional radiation via SBRT after whole-lung irradiation (WLI) of 1500 cGy in 10 fractions. For lesions treated with curative/definitive intent, median dose delivered was 40 Gy (range, 30–60 Gy), delivered in a median of 5 fractions (range, 3–10) of a median of 7.5 Gy per fraction (range, 6–10 Gy). For lesions treated with palliative intent, median dose delivered was 40 Gy (range, 16–50 Gy), delivered in a median of 5 fractions (range, 1–10) of a median of 8 Gy per fraction (range, 5–21 Gy).

**Figure 1:**
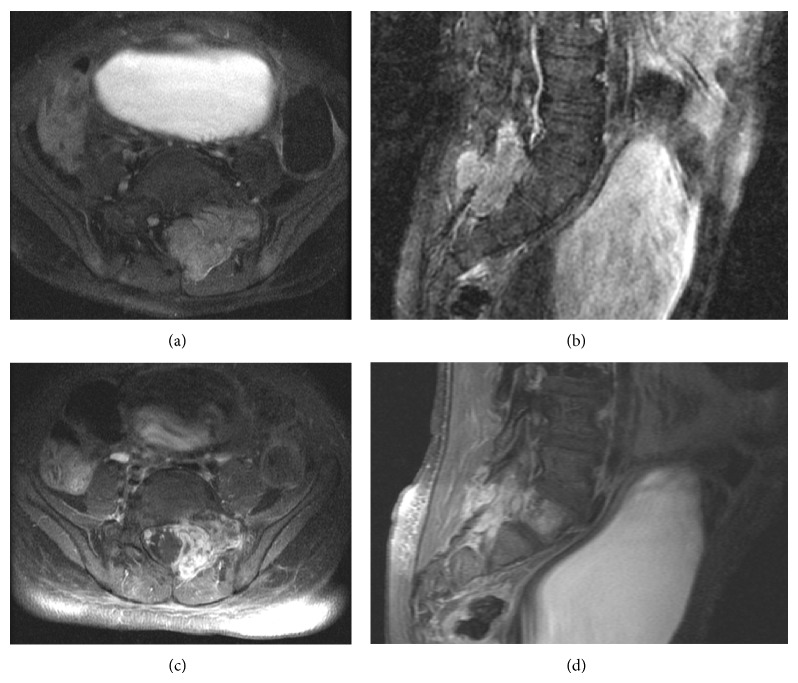
Magnetic resonance images of a sacral osteosarcoma metastasis in a 5-year-old boy. Axial (a and c) and sagittal (b and d) T1-weighted images acquired with gadolinium before treatment (a and b) and after treatment (c and d).

**Figure 2:**
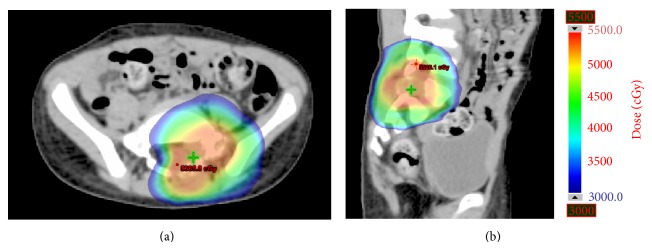
Isodose curves. Axial (a) and sagittal (b) representative isodose curves from the treatment plan used for the osteosarcoma lesion depicted in Figure 1.

Representative pretreatment and posttreatment MRIs and treatment isodose curves for a 5-year-old patient treated with 50 Gy in 5 fractions for a sacral metastasis from osteosarcoma are shown in Figures 1 and 2.

For surviving patients treated curatively or definitively, median follow-up was 2.0 years (range, 1.2–4.0 years). Median follow-up with imaging was 2.0 years (range, 0.7–4.0 years). Of patients treated with curative or definitive intent, one patient had failure in 2 sites after each was treated with 30 Gy in 3 fractions for osteosarcoma. Estimated local control at 2 years was 85%.

For patients treated with palliative intent, median follow-up was 0.2 years (range, 0.04–1.2). Three lesions treated with palliative intent progressed after SBRT. One patient had local progression in 2 locations, after treatment with 40 Gy in 5 fractions and 50 Gy in 10 fractions, respectively, for osteosarcoma. This was felt to be marginal failure/progression of disease that was too large to entirely encompass safely at the time of SBRT. Despite progression, the patient experienced pain improvement. A second patient had failure at a single site after treatment with 25 Gy in 5 fractions for ES. He also experienced pain improvement following treatment.

### 3.2. Toxicity

Early and late toxicity are reported in Tables 2 and 3. No grade 3 or greater acute toxicity was reported. Significant symptomatic late toxicity was seen in 3 patients, although only 1 met criteria for classification as grade 3 toxicity. Grade 3 sacral plexopathy occurred in a patient reirradiated with curative intent, with 60 Gy in 10 fractions to the sacrum 1.75 years after receiving 59.4 Gy in 33 fractions for ES. Symptoms appeared approximately 5 months after SBRT administration. Given that portions of the sacral nerves and plexus were involved by tumor, they were not listed as organs at risk with constraints. During the initial treatment, much of the involved sacral plexus received 105% of the prescription dose. During reirradiation, the involved sacral nerve roots and plexus received 100% of prescription.

**Figure 3:**
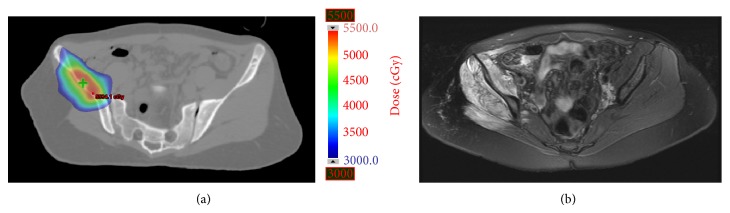
Representative axial dose color wash (a) for a 17-year-old female treated with 50 Gy in 5 fractions for oligometastatic osteosarcoma of the iliac wing and (b) axial magnetic resonance imaging two months after treatment, demonstrating myonecrosis, with correlation between treatment volumes and subsequent soft tissue changes.

Myonecrosis (grade 2 myositis) occurred in one patient 2 months after treatment with 50 Gy in 5 fractions to the right iliac wing with concurrent gemcitabine for osteosarcoma.  Figure 3 shows isodose curves from her SBRT treatment and a posttreatment MRI, demonstrating clear correlation between treatment volumes and subsequent myonecrosis. The patient experienced significant pain and transient paraesthesia as a result. Two years after treatment, her paraesthesia has resolved and her pain is controlled with a narcotic-containing pain regimen.

The third significant late toxicity was in a patient treated with 60 Gy in 10 fractions to an osteosarcoma metastasis of the femoral head. The entirety of the femoral head and neck was involved by disease and received the prescription dose. Four and 8 months after SBRT, he developed grade 2 pathologic fracture and avascular necrosis of the femoral head, respectively. Both were managed conservatively and have not required surgical intervention.

### 3.3. Long-Term Follow-Up

Six patients treated with curative or definitive intent have been followed for more than 1 year after SBRT. One patient is 4 years from treatment for metastatic ES. He received an SBRT boost to T11, with 30 Gy in 5 fractions, after completing WLI. Eighteen months after SBRT, he had disease recurrence in the lung, underwent metastasectomy, and now has no evidence of recurrent ES. He developed myelodysplastic syndrome approximately 3 years after completing his initial ES therapy. A second patient was treated with SBRT for a hilar osteosarcoma metastasis with 50 Gy in 5 fractions and has been followed for 3.5 years with no evidence of recurrence in the treated site, although she had progression elsewhere 21 months after treatment. She had no toxicity from radiotherapy. The third patient is described above, in whom myositis developed after treatment for metastatic osteosarcoma of the iliac wing. At 2 years from treatment, she has no evidence of recurrent disease. The fourth patient with long-term follow-up underwent treatment of three thoracic spine metastases from ES, receiving a 35 Gy boost in 5 fractions after WLI. At last follow-up, 2 years after treatment, he had no evidence of disease and no toxicity. The patient with recurrent Ewing sarcoma of the sacrum, described above, has also been followed up for 2 years. Sacral plexopathy is his only reported toxicity. He developed lung metastases 1.5 years after SBRT. Lastly, the patient treated with 60 Gy in 10 fractions for a femoral head osteosarcoma metastasis has lived 1.6 years, with no evidence of recurrent disease. He experienced avascular necrosis and pathologic fracture due to SBRT, as described above.

One patient treated with palliative intent has survived more than one year. The patient was treated with SBRT to osseous metastases from osteosarcoma in T8 and the left ischium, receiving 4000 cGy in 5 fractions to each. At last follow-up, 14 months after treatment, he had progression elsewhere, but the treated lesions were stable and he had no evidence of toxicity.

## 4. Discussion

Stereotactic body radiotherapy is a theoretically attractive local treatment modality in certain patients with recurrent or metastatic ES and osteosarcoma, as it is convenient, minimizes delays in chemotherapy administration, and offers the possibility of increased efficacy via biologically effective dose escalation. Careful consideration of potential toxicity is warranted, however, particularly in patients with limited life expectancy.

The importance of local therapy for patients with metastatic Ewing sarcoma and the possibility of durable cure in certain patients with metastatic disease have recently been described. Haeusler and colleagues [4] analyzed the effects of local therapy to the primary disease site and sites of metastatic disease for patients with primary, extrapulmonary, disseminated, multifocal ES. They demonstrated that event-free survival was superior in patients who received local therapy—in the form of conventionally fractionated radiotherapy, surgery, or a combination thereof—to all sites of disease involvement, in addition to systemic treatment. On the basis of these findings, patients with primary disseminated ES should be considered for local treatment to the primary lesion and all sites of metastasis, when deemed safe and appropriate in the patient's clinical context. In this series, 4 patients with ES were treated with definitive/curative SBRT, none of whom experienced local failure, three of whom are alive (at 1.6–4 years after treatment), and two of whom are alive with no evidence of disease at 2 and 4 years after SBRT.

Osteosarcoma, unlike ES, has traditionally been considered a radioresistant tumor. In a study of conventional radiotherapy in the primary setting, DeLaney and colleagues [9] found that patients treated with radiotherapy after biopsy only, with doses equivalent to 68 Gy or higher in 2 Gy fractions, had only 40% local control at 5 years. Local control was slightly, although not significantly, better with higher radiation dose. Based on these and other similar data, radiotherapy has been reserved for tumors not amenable to surgical resection. In order to provide patients without feasible surgical options with a more effective alternative, recent interest has turned to the possible benefit of hypofractionation, which theoretically could overcome the reported radioresistance of osteosarcoma. Matsunobu and colleagues [10] studied patients with unresectable osteosarcoma of the trunk treated with carbon therapy. The median delivered dose was 70.4 Gy equivalents in 16 fractions over 4 weeks; the resulting 5-year local control rate was 62%. Patients with tumors smaller than 500 cm^3^ had an 88% local control rate.

Given historically poor local control with conventional radiotherapy for osteosarcoma and the suggestion of dose-response, the possibility of dose escalation in the treatment of osteosarcoma is appealing. Similarly, in light of the recent data of Matsunobu and colleagues [10] (recognizing inherent differences in physical properties of photons versus carbon ions), hypofractionation is increasingly thought to be beneficial in improving local control. Consequently, treatment modalities that allow for safe delivery of high-dose, hypofractionated radiotherapy offer promise in patients with osteosarcoma who are not surgical candidates. In this series, 5 of 6 patients (5 of 7 lesions) treated with SBRT with definitive/curative intent for osteosarcoma did not experience local failure. The patient who had local relapse of disease was treated with SBRT for two large sarcomatous lesions arising in a previously radiated field. It is possible that the large size of the tumors treated and inherent aggressiveness of a possible secondary malignancy contributed to failure of SBRT to control this disease. Three of the six patients treated definitively died within 1 year of SBRT receipt, all of whom had multiple sites of disease at the time of SBRT, underscoring the importance of proper patient selection and avoidance of toxicity for patients with limited lifespan. However, three of six patients, all of whom had a single site of disease at the time of SBRT, have had durable (1.6–3.5 years) local control of treated lesions, and two are without evidence of recurrent disease, suggesting that SBRT is a viable consideration for select patients.

In addition to potential use in the curative or definitive treatment of metastatic and recurrent ES and osteosarcoma, radiotherapy plays an important role in the palliation of symptoms related to these diseases. Even in osteosarcoma, radiation has long been used for palliation of symptoms in patients with metastatic disease, with good results in terms of pain reduction and mediocre durable control of disease at the treated site [11]. SBRT was effective in palliating symptoms in this series; however, most patients treated with palliative intent died of disease within 6 months of receiving SBRT. Cost, expected outcome, life expectancy, and benefit of SBRT versus conventional radiation should be considered before palliative SBRT is offered.

This observational series provides important instructive toxicity data that underscores the potential risks of SBRT. Three patients in this study experienced significant late toxicity. Two of these patients were treated with doses beyond those recommended by TG-101. Only 1 patient treated in a manner respecting TG-101 [12] constraints (with dose reduction as described above) experienced significant toxicity. This highlights the importance of respecting normal tissues when possible and proper patient counseling when constraints are knowingly exceeded. One patient developed grade 3 sacral plexopathy after high-dose reirradiation for localized, recurrent ES. For most pelvic SBRT treatments that pose risk to the sacral plexus, TG-101 [12] dose constraints for the nerve roots and plexus are respected, in order to reduce the risk of neuropathy. However, in this particular case, violation of constraints was allowed, for fear of treatment failure if volumes and doses were reduced, particularly given prior progression with conventionally fractionated radiotherapy. Thoughtful and candid consent was obtained from the patient, who felt that the risk of substantial neuropathy was preferable to hemipelvectomy, the surgical alternative to proceeding with reirradiation. Similarly, the patient with femoral head necrosis and fracture was counseled regarding the potential risk of toxicity and accepted the risk over surgical extirpation of disease.

One patient whose treatment met requested dose limits experienced myonecrosis after SBRT. This was likely due to concurrent administration of gemcitabine. Radiation recall after gemcitabine administration has been previously described [13]. Specific reactions reported in the literature vary widely but are universally of inflammatory etiology. Timing of the reaction is variable, with reports of symptomatic radiation recall several weeks to many months after administration of gemcitabine. Knowledge of this possible toxicity allows for appropriate counseling.

This study is, at the time of this writing, the first report of techniques and outcomes with SBRT for recurrent and metastatic ES and osteosarcoma. The study is limited by the small number of patients included and by short follow-up. Prior reports have demonstrated that longer follow-up is necessary to observe local recurrences following SBRT relative to conventional radiotherapy. Therefore, given the short follow-up in our report, it is not possible to draw meaningful conclusions regarding local control outcomes. Long-term follow-up is also critical to fully capture late toxicity associated with treatment. Although we report follow-up of greater than a year for 7 patients, toxicities can occur later than one year after SBRT, and thus no definitive conclusions regarding occurrence rates of long-term toxicities should be drawn.

The toxicities reported herein can help guide patient selection for SBRT going forward. This report underscores the importance of respecting SBRT dose constraints when feasible and careful patient counseling when constraints are exceeded. It also illustrates the importance of careful patient selection and counseling, bearing in mind expected duration of survival as well as collaboration with the multidisciplinary team to coordinate chemotherapy timing and agent choice.

Most importantly, this small series demonstrates the need for systematic study of the use of SBRT in pediatric sarcomas such as ES and osteosarcoma. The Children's Oncology Group is proposing a protocol (DuBois S, written communication, June 2013) to assess the feasibility of administering SBRT to patients with metastatic ES in the context of a cooperative group trial. The protocol will use a dose of 40 Gy in 5 fractions and will provide very valuable information regarding toxicity and effectiveness of SBRT for ES. Such standardized trials are the cornerstone of evaluation of evolving treatment modalities.

## 5. Conclusion

In conclusion, SBRT is a theoretically useful modality of radiation delivery for patients with recurrent or metastatic ES and osteosarcoma. More data is necessary before conclusions can be drawn regarding efficacy of treatment. Consideration of patient context and expected longevity is paramount. Significant toxicity may occur when established dose constraints are exceeded.

## Figures and Tables

**Table 1:** Patient and tumor characteristics.

Characteristic	Value^a^
Patients (*n* = 14)	
Age, y	24 (4.9–66.4)
Histology	
Osteosarcoma	9
Ewing sarcoma	5
Disease	
Metastatic	13
Recurrent, localized	1
Lesions treated (*n* = 27)	
Histology	
Osteosarcoma	19
Ewing sarcoma	8
Location	
Osseous	21
Pulmonary/mediastinal	6

^a^Values are median (range) or number of patients.

**Table 2:** Description of definitive treatments^*^.

Histology (solitary versus multiple lesions present)	Patient age at time of SBRT (years)	Concurrent chemotherapy	Location	PTV size (cc)	SBRT total dose (Gy)	Fractions (number)	Local failure (time to failure, years)	Overall disease status (F/U, years)	Time to F/U imaging^1^ (years)	Acute toxicity (grade)	Late toxicity (grade)
**Metastatic OGS (solitary)^2^**	**17.7**	**Gemcitabine and docetaxel**	**Iliac wing**	**13**	**50**	**5**	**No**	**NED (2.0)**	**2.0**	**None**	**Myonecrosis (2), pain (2), neuropathy (2)**
Metastatic OGS (solitary)	9.8	Ifosfamide and etoposide	Femoral head	43	60	10	No	NED (1.6)	1.6	Erythema (1)	AVN (2), pathologic fracture (2)
**Metastatic OGS (solitary)**	**23.8**	**None**	**Hilum**	**24**	**50**	**5**	**No**	**AWD (3.5)**	**3.5**	**None**	**None**
Metastatic OGS (multiple)	66.4	None	S1	175	30	3	Yes (0.4)	DOD (0.8)	0.4	None	None
Metastatic OGS (multiple)^2^	66.4	None	L3	99	30	3	Yes (0.1)	DOD (0.8)	0.1	None	None
**Metastatic OGS (multiple)**	**17.3**	**None**	**Iliac crest**	**11**	**60**	**10**	**No**	**DOD (0.6)**	**None**	**None**	**None**
Metastatic OGS (multiple)	4.9	None	Sacrum	18	50	5	No	DOD (0.3)	0.2	None	None
**Recurrent ES (solitary)** ^ 3^	**23.1**	**None**	**Sacrum**	**91**	**60**	**10**	**No**	**AWD (2.0)**	**2.0**	**None**	**Neuropathy (3)**
Metastatic ES (multiple)^4^	15.9	None	T11	5.6	30	5	No	NED (4.0)	4.0	None	Myelodysplastic syndrome
**Metastatic ES (multiple)^5^**	**19.7**	**Vincristine, topotecan, and cytoxan**	**RUL**	**1.5**	**40**	**5**	**No**	**DOD (0.6)**	**0.3**	**None**	**None**
**Metastatic ES (multiple)^5^**	**19.7**	**Vincristine, topotecan, and cytoxan**	**RLL**	**2.3**	**40**	**5**	**No**	**DOD (0.6)**	**0.3**	**None**	**None**
Metastatic ES (multiple)^4^	30.2	Ifosfamide and etoposide	T1-2	0.7	35	5	No	NED (2.0)	2.0	None	None
Metastatic ES (multiple)^4^	30.2	Ifosfamide and etoposide	T5	0.7	35	5	No	NED (2.0)	2.0	None	None
Metastatic ES (multiple)^4^	30.2	Ifosfamide and etoposide	T12-L2	4.8	35	5	No	NED (2.0)	2.0	None	None

^*^Continguous similarly bolded/non-bolded rows denote multiple lesions in an individual patient.

^1^Time from SBRT to last follow-up imaging.

^2^Treatment volume overlapped with field previously irradiated to 40 Gy in 20 fractions for plasmacytoma 6 years before.

^3^Retreatment of recurrent disease, following 59.4 Gy in 33 fractions to the same region.

^4^SBRT delivered immediately following whole-lung irradiation to 15 Gy in 10 fractions.

^5^Status after contralateral pneumonectomy.

PTV: planning target volume; SBRT: stereotactic body radiotherapy; OGS: osteosarcoma; NED: no evidence of disease; DOD: dead of disease; ES: Ewing sarcoma; RUL: right upper lobe of lung; RLL: right lower lobe of lung; AWD: alive with disease.

**Table 3:** Description of palliative treatments^*^.

Histology^1^	Patient age at time of SBRT (years)	Concurrent chemotherapy	Location	PTV size (cc)	SBRT total dose (Gy)	Fractions (number)	Pretreatment symptoms	Symptom relief^2^	Overall disease status (F/U, years)	Acute toxicity (grade)	Late toxicity (grade)
**OGS**	**17.7**	**None**	**Scapula**	**54**	**21**	**1**	**Pain**	**Complete, durable**	**DOD (0.2)**	**None**	**N/A**
**OGS**	**17.7**	**None**	**C7-T1**	**34**	**24**	**3**	**None**	**N/A**	**DOD (0.2)**	**None**	**N/A**
**OGS**	**17.7**	**None**	**T4**	**17**	**24**	**3**	**Pain**	**Unclear^3^**	**DOD (0.2)**	**None**	**N/A**
OGS	5.1	None	T7–9	8.5	16	1	Pain	Complete, durable	DOD (0.04)	None	N/A
**OGS^4^**	**24.2**	**Topotecan and cyclophosphamide**	**Left lung**	**432**	**50**	**10**	**Pain**	**Partial, transient**	**DOD (0.8)**	**Esophagitis (2)**	**None**
**OGS**	**24.5**	**None**	**Mediastinum**	**127**	**50**	**10**	**Hemoptysis**	**Durable**	**DOD (0.5)**	**None**	**None**
**OGS^5^**	**24.7**	**None**	**Left lung**	**67**	**40**	**5**	**Pain**	**Partial, transient**	**DOD (0.3)**	**Pain flare (2)**	**None**
**OGS**	**24.9**	**None**	**Zygoma**	**2.4**	**16**	**1**	**Pain**	**Complete, durable**	**DOD (0.1)**	**None**	**N/A**
OGS	33.4	None	T8	10	40	5	None	N/A	AWD (1.2)	None	None
OGS	33.4	None	Ischium	9.6	40	5	None	N/A	AWD (1.2)	None	None
**ES**	**28.7**	**None**	**Sacrum and left ilium**	**891**	**50**	**5**	**Pain**	**Complete, durable**	**DOD (0.1)**	**None**	**N/A**
**ES**	**28.7**	**None**	**C2**	**24**	**50**	**10**	**Pain**	**Complete, durable**	**DOD (0.1)**	**None**	**N/A**
ES^6^	63.9	None	L2	3.9	25	5	Pain	Partial, transient	DOD (0.3)	None	None

^*^Continguous similarly bolded/non-bolded rows denote multiple lesions in an individual patient.

^1^All patients had metastatic disease.

^2^Durable: until death or last follow-up.

^3^Intrathecal pain pump placed immediately after SBRT; pain relief ensued.

^4^Local failure noted at 0.6 years.

^5^Local failure noted at 0.1 years.

^6^Local failure noted at 0.2 years.

PTV: planning target volume; SBRT: stereotactic body radiotherapy; OGS: osteosarcoma; DOD: dead of disease; ES: Ewing sarcoma; AWD: alive with disease.
